# Epidemiologic and Ecologic Investigations of Monkeypox, Likouala Department, Republic of the Congo, 2017

**DOI:** 10.3201/eid2502.181222

**Published:** 2019-02

**Authors:** Reena H. Doshi, Sarah Anne J. Guagliardo, Jeffrey B. Doty, Angelie Dzabatou Babeaux, Audrey Matheny, Jillybeth Burgado, Michael B. Townsend, Clint N. Morgan, Panayampalli Subbian Satheshkumar, Nestor Ndakala, Therese Kanjingankolo, Lambert Kitembo, Jean Malekani, Lem’s Kalemba, Elisabeth Pukuta, Tobi N’kaya, Fabien Kangoula, Cynthia Moses, Andrea M. McCollum, Mary G. Reynolds, Jean-Vivien Mombouli, Yoshinori Nakazawa, Brett W. Petersen

**Affiliations:** Centers for Disease Control and Prevention, Atlanta, Georgia, USA (R.H. Doshi, S.A.J. Guagliardo, J.B. Doty, A. Matheny, J. Burgado, M.B. Townsend, C.N. Morgan, P.S. Satheshkumar, A.M. McCollum, M.G. Reynolds, Y. Nakazawa, B.W. Petersen);; Ministry of Health, Brazzaville, Democratic Republic of the Congo (A.D. Babeaux, L. Kitembo, F. Kangoula, J.-V. Mombouli);; Oak Ridge Institute for Science and Education, Centers for Disease Control and Prevention Fellowship Program, Oak Ridge, Tennessee, USA (A. Matheny, J. Burgado, C.N. Morgan);; Centers for Disease Control and Prevention, Kinshasa, Democratic Republic of the Congo (N. Ndakala, T. Kanjingankolo, J.-V. Mombouli);; University of Kinshasa, Kinshasa (J. Malekani, L. Kalemba);; Institut Nationale de Recherche Biomedicale, Kinshasa (E. Pukuta); Ministere de l’Agriculture, de l’Elevage et de la Peche, Brazzaville (T. N’kaya);; International Communication and Education Fund, Kinshasa (C. Moses)

**Keywords:** orthopoxvirus, Republic of the Congo, zoonoses, monkeypox, viruses, ecology, epidemiology

## Abstract

Monkeypox, caused by a zoonotic orthopoxvirus, is endemic in Central and West Africa. Monkeypox has been sporadically reported in the Republic of the Congo. During March 22–April 5, 2017, we investigated 43 suspected human monkeypox cases. We interviewed suspected case-patients and collected dried blood strips and vesicular and crust specimens (active lesions), which we tested for orthopoxvirus antibodies by ELISA and monkeypox virus and varicella zoster virus DNA by PCR. An ecologic investigation was conducted around Manfouété, and specimens from 105 small mammals were tested for anti-orthopoxvirus antibodies or DNA. Among the suspected human cases, 22 met the confirmed, probable, and possible case definitions. Only 18 patients had available dried blood strips; 100% were IgG positive, and 88.9% (16/18) were IgM positive. Among animals, only specimens from *Cricetomys* giant pouched rats showed presence of orthopoxvirus antibodies, adding evidence to this species’ involvement in the transmission and maintenance of monkeypox virus in nature.

Monkeypox virus (MPXV) is a zoonotic orthopoxvirus, endemic to the heavily forested regions of West and Central Africa. MPXV is a close relative of variola virus (the causative agent of smallpox), and its clinical presentation resembles smallpox, with the addition of lymphadenopathy (*1*–*3*). Case-fatality rates have been reported to be as high as 11% (*4*). Vaccination with a traditional smallpox vaccine has been shown to be protective for monkeypox, but since the eradication of smallpox in 1980, routine smallpox vaccination has ceased (*3*,*5*). The incidence of human monkeypox appears to have increased in countries to which the virus is endemic; it is unknown whether this increase is the result of waning population-level immunity or other factors (*6*–*8*).

Humans can acquire MPXV via respiratory droplets or other bodily fluids or by direct contact with lesion material of infected patients (*9*). Zoonotic transmission may occur by direct inoculation via bites and scratches (*10*) or direct contact with bodily fluids of infected animals when hunting, preparing carcasses for meals, or playing with animals (*4*,*11*). The wild animal reservoir of MPXV remains unknown; however, evidence implicates rodents and other small mammals (*12*–*15*), whereas infection of humans and monkeys appears to be incidental (*16*). To date, live MPXV from wildlife species has been isolated only from a rope squirrel (*Funisciurus anerythrus*) (*12*) and a sooty mangabey (*Cercocebus atys)* (*17*).

More than 90% of monkeypox cases occur within the Congo Basin, with the largest number of cases reported in the Democratic Republic of the Congo (DRC) (*11*,*18*,*19*). Monkeypox cases have only sporadically been reported in the neighboring Republic of the Congo, generally in remote, heavily forested areas near the border with DRC, where humans are frequently in contact with animals. Previous monkeypox outbreaks occurred in Likouala Department, in the northern part of Republic of the Congo, in 2003, 2007, and 2010, in which the agent was closely related to MPXV found in DRC (*20*,*21*).

In January 2017, two suspected monkeypox cases were reported in Moualé village in Likouala Department of Republic of the Congo. By March 8, seven additional suspected cases had been reported. We report on the investigation and analysis of human monkeypox cases reported during January–April 2017 (and further investigated during March 22–April 5, 2017) in this area, including epidemiologic description, factors associated with disease acquisition, and a subsequent ecologic investigation of possible zoonotic sources.

## Materials and Methods

### Study Sites and Description of Outbreak

**Figure 1:**
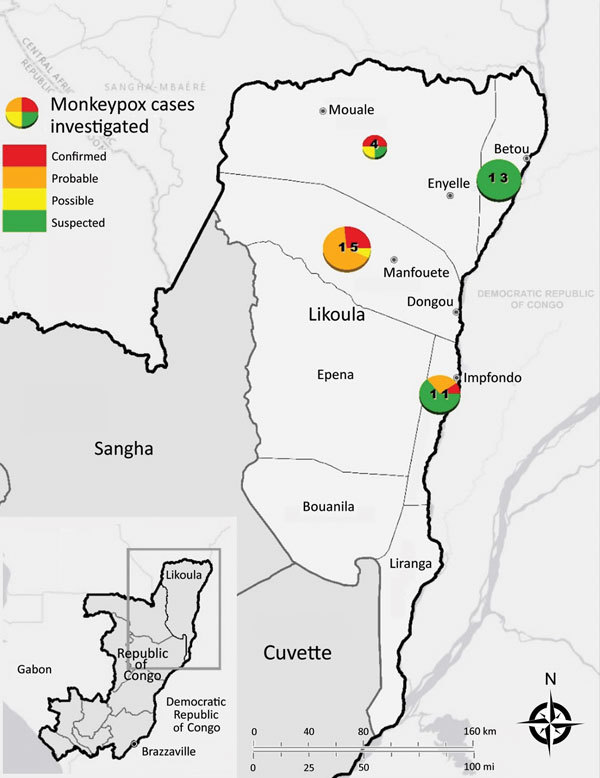
Locations of monkeypox outbreaks and case classifications, Likouala Department, Republic of Congo, 2017. Numbers in circles indicate total number of cases in each area (all case classifications). Inset shows location of study area within Republic of Congo.

This outbreak investigation involved suspected monkeypox cases from 4 districts, Impfondo, Betou, Dongou, and Enyelle, in Likouala Department. The department is 1 of 12 administrative regions located in northeastern Republic of the Congo and has ≈154,000 residents (Figure 1) (*22*). The largest town, Impfondo, is the administrative capital and is located ≈185 km south of Betou. The department is divided into 7 districts and is characterized by dense tropical rainforest (*23*). Most of the inhabitants rely on subsistence agriculture (cassava, corn, plantains), fish, and consumption of bushmeat and insects (e.g., antelopes, wild pigs, pangolins, small deer, porcupines, small rodents, primates, crocodiles, tortoises, snails, caterpillars) as sources of nutrition.

On January 21, 2017, a 40-year-old fisherman and his 9-year-old son sought care for fever and rash at a local health center in Moualé, Enyelle District. The reported onset of fever was January 18 for the fisherman and January 20 for his son. The fisherman died on February 25 from unknown complications. On January 27, 2017, the General Direction of Epidemiology and Disease Control in Brazzaville, Republic of the Congo, was notified of the 2 suspect monkeypox cases, and an investigation was conducted on January 29. MPXV nucleic acid was detected by PCR in crust/vesicle specimens from both cases on February 13 at the Institut Nationale de Recherche Biomedicale in Kinshasa, DRC. By March 7, an additional 8 suspected cases were reported in Likouala. At the same time, there were also reports of a large measles outbreak in Betou and Enyelle Districts. A multipartner epidemiologic investigation occurred during March 22–April 4, 2017.

### Epidemiologic and Clinical Information

Active and retrospective cases were identified and reported by health facilities, patients, and family/community members on the basis of clinical signs and symptoms. Confirmed and suspected monkeypox cases were investigated and data were collected using the Ministry of Health’s standardized case report form. In addition, physician notes and hospital records were reviewed when available. An additional risk factor survey was administered to both confirmed and suspected case-patients to identify possible sources of monkeypox exposure during the 4 weeks before symptom onset. For young children and deceased patients, a parent, guardian, or other family member was interviewed. When possible, we constructed transmission chain diagrams to document the spread of disease throughout the community.

Case definitions were adapted from previous investigations and were applied to cases investigated during January 1–April 2, 2017 (Table 1) (*24*). A suspected case was defined as unexplained rash and fever (subjective or measured temperature of >99.3°F [>37.4°C]). A possible case met 1 of the epidemiologic criteria or demonstrated elevated levels of orthopoxvirus-specific IgM and had unexplained rash and fever and >2 other signs or symptoms from the clinical criteria. A probable case met 1 of the epidemiologic criteria and demonstrated elevated levels of orthopoxvirus-specific IgM and had unexplained rash and fever and >2 other signs or symptoms from the clinical criteria. A confirmed case involved detection of orthopoxvirus DNA by PCR testing of a clinical specimen (laboratory criterion).

**Table 1:** Criteria for monkeypox case classification, Likouala, Republic of the Congo, 2017

Criteria
Clinical
Rash: muscular, papular, vesicular, pustular, generalized, or localized; discrete or confluent
Fever >37.4°C
Chills and/or sweats
Headache
Backache
Lymphadenopathy
Sore throat/cough
Coryza
Malaise
Prostration/distress
Epidemiologic
Exposure to a confirmed or probable human case of monkeypox
Exposure to an African endemic animal species of which cases have been identified with elevated levels of orthopoxvirus
Laboratory
Detection of orthopoxvirus DNA by PCR testing of a clinical specimen
Exclusion
No detection of orthopoxvirus DNA by PCR testing of a well-sampled rash lesion
Alternative diagnosis can fully explain the illness

### Specimen Collection and Laboratory Analysis

We collected 2 lesion specimens (crust or vesicle swabs) from case-patients with active rash. In addition, we collected dried blood strips from active suspected case-patients, retrospective suspected case-patients, and household contacts who were available and willing to participate.

Swab and crust specimens were tested at the Institut Nationale de Recherche Biomedicale for orthopoxvirus and, if negative, for varicella zoster virus DNA signatures by PCR. Dried blood strips were tested at CDC (Atlanta, GA, USA) for orthopoxvirus IgG and IgM by ELISA, as previously described (*25*). All dried blood strip specimens were tested at dilutions of 1:100 or 1:50 in each ELISA while accounting for additional dilution after elution from the dried blood strips.

### Surveillance Strengthening

During the investigation, healthcare workers in the affected areas received training in monkeypox clinical characteristics and case recognition, case management, surveillance, patient care, and infection prevention and control (*26*). In addition, the International Communication and Education Foundation provided assistance with community education by screening educational videos, broadcasting radio messages, and facilitating discussions with community members (*26*).

### Ecologic Investigation

We conducted an ecologic investigation of wildlife in and around the village of Manfouété in Dongou District in August 2017, five months after the epidemiologic investigation. Manfouété is a small village on the Motaba River. The village has ≈1,000 inhabitants of both Bantu and Autochthon (Pygmy) ethnic groups. The largest number of suspected cases was reported in Manfouété, which is surrounded by dense, moist forest and edaphic forest (*27*).

For the investigation, we captured small mammals using live traps, snap traps, and pitfall traps to increase the diversity of the sample. Traps were placed in forested areas, heavily disturbed sites, and in homes around the village. All captured animals were transported to a central processing area, where they were anesthetized with halothane or ketamine and then humanely euthanized following CDC Institutional Animal Care and Use Committee approved protocols (DOTMULX2660). Standard measurements (total length, tail length, right hind foot length, ear length, weight) were recorded to help with species identification. The animals were assessed for wounds and poxvirus-like lesions. We collected a variety of specimens from each animal (serum, dried blood, liver, spleen, heart, lung, and kidney). All specimens were stored in liquid nitrogen and shipped to CDC in Atlanta for processing.

Serum and dried blood specimens were assessed by modified ELISA for anti-orthopoxvirus IgG antibodies in specimen dilutions of 1:100, 1:200, and 1:400, as previously described (*15*,*25*). An animal was confirmed positive for the presence of orthopoxvirus antibodies if the specimen optical density value was above the cutoff values in >2 consecutive dilutions (1:100 and 1:200). Tissue specimens were tested for the viral DNA by PCR. DNA extraction was conducted with the MagMAX magnetic particle extraction robot (ThermoFisher, https://www.thermofisher.com), and the presence of viral DNA was assessed using real-time PCR to detect the E9L gene of orthopoxvirus (*28*). A 400-bp fragment of the mitochondrial *cytochrome B* (*cytB*) gene was amplified for the 9 *Cricetomys* giant pouched rat specimens collected in the Manfouété area using primers MVZ05 (*29*) and R400 (*30*). The purified PCR products were then sequenced on an ABI PRISM 3130-Avant Genetic Analyzer (Applied Biosystems, https://www.thermofisher.com). Sequences were assembled and proofed in Geneious version 10.2.2 (https://www.geneious.com). Representative sequences of the 6 *Cricetomys* rat species proposed by Olayemi et al. (*31*) were selected to conduct a Bayesian inference analysis using MrBayes version 3.2.6 (*32*,*33*) to confirm the megaBLAST (https://blast.ncbi.nlm.nih.gov/Blast.cgi) species identification of these specimens.

## Results

We investigated 43 suspected cases from March 22–April 5, 2017, in Betou, Enyelle, Impfondo, and Manfouété. We also interviewed 11 household contacts who provided dried blood strips. We collected specimens from crust, vesicles, or both from 4 active suspected case-patients in Manfouété.

Among the suspected cases, 7 met the confirmed case definition, 13 met the probable case definition, and 2 met the possible case definition (Table 2). We excluded 6 cases and investigated an additional 15 suspected cases but were unable to classify them because of a missing case report form, missing exposure information, the inability to match specimens with the case report form, or some combination.

**Table 2:** Exposure histories, illness characteristics, laboratory results, and outcomes for confirmed, probable, and possible monkeypox cases, Likouala Department, Republic of the Congo, 2017

Case no.	Case category	Patient age, y/sex	Onset date	Orthopoxvirus*			District†	Outcome‡	Exposure
				IgG	IgM	PCR			
1e	Confirmed	40/M	Jan 18	No specimen	No specimen	Positive	Enyelle	Dead	Unknown
2e	Confirmed	9/M	Jan 20	Unable to link	Unable to link	Positive	Enyelle	Alive	Son of 1e
3e	Possible	24/M	Mar 21	Positive	Positive	No specimen	Enyelle	Alive	Unknown
1d	Confirmed	12/F	Jan 28	Positive	Positive	Positive	Dongou	Alive	Hunter
2d	Confirmed	4/F	Feb 12	No specimen	No specimen	Positive	Dongou	Dead	Contact with suspected case (never located)
3d	Confirmed	28/F	Feb 25	Positive	Positive	Positive	Dongou	Alive	Hunter/bushmeat merchant, contact with 1d
4d	Confirmed	20/F	Mar 15	Positive	Positive	Positive	Dongou	Alive	Hunter, contact with 1d, 3d, 15d
5d	Probable	33/F	Mar 24	Positive	Positive	Collected, lost	Dongou	Alive	Mother of 2d
6d	Probable	3/F	Mar 30	Positive	Positive	No specimen	Dongou	Alive	Family cluster, contact with 7d, 8d, 10–14d
7d	Probable	1/F	Unknown	Positive	Negative	No specimen	Dongou	Alive	Family cluster, contact with 6d, 8d, 10–14d
8d	Probable	30/M	Unknown	Positive	Positive	Not collected	Dongou	Alive	Hunter/family cluster, contact with 6d, 7d, 10–14d
9d	Possible	27/F	Unknown	Positive	Positive	Not collected	Dongou	Alive	Unknown
10d	Probable	11/M	Unknown	Positive	Positive	No specimen	Dongou	Alive	Hunter/family cluster, contact with 6–8d, 11–15d
11d	Probable	15/F	Unknown	Positive	Negative	No specimen	Dongou	Alive	Hunter/family cluster, contact with 6–8d, 10d, 12–14d
12d	Probable	5/F	Unknown	Positive	Positive	No specimen	Dongou	Alive	Family cluster, contact with 6–8d, 10d, 11d, 13d, 14d
13d	Probable	5/F	Unknown	Positive	Positive	No specimen	Dongou	Alive	Family cluster, contact with 6–8d, 10–12d, 14d
14d	Probable	28/F	Unknown	Positive	Positive	Not collected	Dongou	Alive	Hunter/family cluster, contact with 6–8d, 10–13d
15d	Probable	1/M	Unknown	Positive	Positive	Not collected	Dongou	Alive	Child of 3d
1i	Probable	14/F	Feb 2	No specimen	No specimen	No specimen	Impfondo	Dead	Prepared bush meat for food (rats, rodents); sister of 2i–4i; first family member to fall ill
2i	Confirmed	11/F	Feb 24	Positive	Positive	Positive	Impfondo	Alive	Sister of 1i, 3i, 4i
3i	Probable	3/M	Mar 4	Positive	Positive	No specimen	Impfondo	Alive	Brother of 1i, 2i, 4i
4i	Probable	8/M	Mar 20	Positive	Positive	Collected, lost	Impfondo	Alive	Brother of 1i, 2i, 3i

*Unable to link is defined as an inability to match specimens with the case report form because of coding errors; no specimen means that a specimen was never collected.  †All case-patients in Dongou were from Manfouété village. ‡Outcome is defined as patient status at the time of the investigation.

Among the 22 confirmed, probable, and possible cases, the median age of patients was 11.5 years (range 1–40 years), and 14 (63.6%) were female. Three were from Enyelle, 15 from Manfouété in Dongou (2 were seen at the Impfondo Base Hospital), and 4 were from Impfondo. Three investigated case-patients were deceased at the time of the investigation. For the 22 cases we analyzed, 18 patients had available dried blood strips; all 18 (100%) were IgG positive and 88.9% (16/18) were IgM positive.

### Enyelle

Two of the 3 cases investigated in Enyelle were confirmed, and 1 met the possible case definition. The possible case-patient was a 24-year-old man whose samples tested positive for orthopoxvirus IgG and IgM. It is unknown whether he was in contact with either confirmed case-patient.

### Dongou

We investigated 15 cases in Dongou District, all in Manfouété. All investigated cases were autochthonous. Investigations revealed that the likely first case was in a male autochthon hunter from the Dinyonga/Bimbema camp. Details of the severity or source of his symptoms are unknown. He had been in contact with a 4-year-old girl (case-patient 2d) who developed severe monkeypox and later died in Impfondo. He had also been in contact with most of the other suspected case-patients in Manfouété. On March 24, the 33-year-old full-term pregnant mother (case-patient 5d) of case-patient 2d was admitted to the Impfondo Base Hospital. She was suspected to have been infected while caring for her daughter. Serologic results indicated positivity for orthopoxvirus IgG and IgM.

Samples from case-patient 1d, a 12-year-old female hunter, were confirmed positive by orthopoxvirus PCR and epidemiologically linked to 2 orthopoxvirus PCR-positive cases (case-patients 3d and 4d) and a probable case with an unknown date of symptom onset (case-patient 15d) that was positive for orthopoxvirus IgM and IgG.

Case-patients 6d–14d were considered probable or possible cases and were all epidemiologically linked; however, the dates of symptom onset were unavailable. Among this cluster, all were orthopoxvirus IgG positive, and all were orthopoxvirus IgM positive except for case-patients 7d and 11d.

### Impfondo

**Figure 2:**
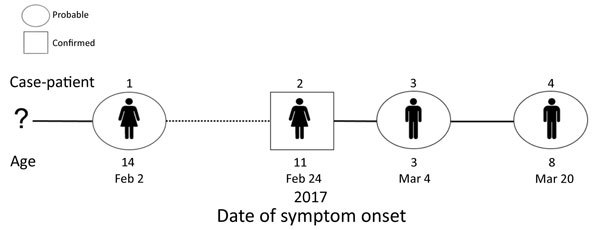
Transmission chain: pattern of virus transmission hypothesized to have occurred during monkeypox outbreak in Impfondo, Likouala Department, Republic of Congo, 2017. Case-patients are arranged according to date of symptom onset. Solid lines indicate probable lines of person-to-person transmission and dashed lines depict undetermined (hypothesized) transmission events. The number of days between onsets (case interval) is approximated by length of lines. Case-patients illustrated are siblings belonging to the same household.

On March 23, an 8-year-old male suspected case-patient (case-patient 4i) was investigated at the Impfondo Base Hospital for a monkeypox-like rash. The boy regularly shared a bed with his siblings, 1 of whom had laboratory-confirmed MPXV infection. Detailed discussions with the family suggested the first case in the family cluster was a 14-year-old girl (case-patient 1i) who developed a fever on February 2, 2017, and a rash 4 days later. On February 8, she died. The family described consuming bushmeat, including small rodents, and case-patient 1i reportedly handled and prepared the rodents before the onset of illness. Three siblings of case-patient 1i subsequently developed similar monkeypox-like symptoms. Fourteen days after the suspected index case-patient’s death, her 11-year-old sister (case-patient 2i) was admitted to the hospital with a rash illness consistent with the suspected case definition. Lesion specimens obtained from this patient were confirmed orthopoxvirus positive by PCR. Eight days after fever onset, her 3-year-old male sibling (case-patient 3i) also developed a fever and subsequently a monkeypox rashlike illness. His symptoms were less severe, and he was never admitted to the hospital. A reconstructed transmission chain is shown in Figure 2. All 7 members of the household were tested; 5 were both IgM and IgG positive for monkeypox, but 1 of these never developed monkeypox-specific signs or symptoms (data not shown).

### Betou

A 12-year-old girl came to the Betou health clinic with a rash illness and fever on March 21, 2017. She was later confirmed PCR negative for monkeypox but was varicella zoster virus positive, suggesting the possibility of a concurrent varicella zoster virus outbreak in the area. We identified and investigated 13 additional suspected cases in the district. The investigation team was also notified of a confirmed measles outbreak in the district. The team indicated that 4/14 cases investigated were students from the same class at 1 school, suggesting the possibility of school-associated monkeypox or measles virus infection or possibly varicella zoster virus with or without monkeypox co-infection.

### Ecologic Investigation

We obtained samples from 105 mammals during 1,843 trap-nights (number of traps per night multipled by the number of nights) over a period of 9 days (Table 3). The mammals sampled belong to the orders Rodentia (77.1%), Eulipotyphla (21.0%), and Chiroptera (1.9%). We found 2 (1.9%) of 105 serum specimens to be positive for orthopoxvirus IgG by ELISA (Table 3). Both of the seropositive animals were rodents of the species *Cricetomys emini* (N = 9, 22.2%). We found all specimens collected in the study to be negative for the presence of orthopoxvirus DNA by real-time PCR.

**Table 3:** Summary of orthopoxvirus IgG ELISA results from mammals sampled in Manfouété, Likouala Department, Republic of the Congo, 2017

Order	Genus	Common name	No. sampled	No. (%) positive
Rodentia	*Hylomyscus*	African wood mouse	28	0
	*Malacomys*	Long-footed rat	15	0
	*Praomys*	Soft-furred rat	15	0
	*Cricetomys*	Giant pouched rat	9	2 (22.2)
	*Hybomys*	Hump-nosed mouse	6	0
	*Rattus*	Rat	3	0
	*Lemniscomys*	Zebra mouse	2	0
	*Mastomys*	Multimammate rat	2	0
	*Atherurus*	Brush-tailed porcupine	1	0
Eulipotyphla	*Sylvisorex*	Climbing shrew	8	0
	*Crocidura*	White-toothed shrew	8	0
	Unknown shrew	Shrew	6	0
Chiroptera	*Pipistrellus*	Pipistrelle bat	1	0
	*Epomops*	Singing fruit bat	1	0
Total			105	2 (1.9)

**Figure 3:**
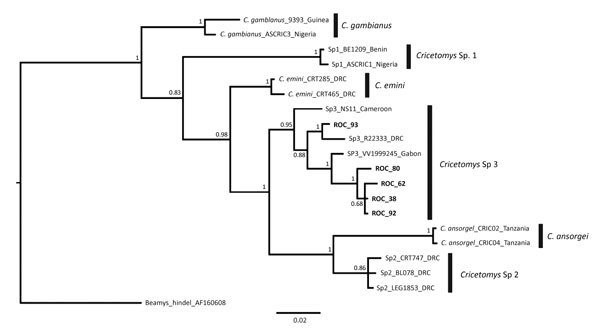
Bayesian majority rules consensus tree comparing sequences obtained from *Cricetomys* specimens collected in Likouala Department, Republic of the Congo, 2017 (boldface), with sequences from Olayemi et al. (*31*). Vertical black bars distinguish clades representing *Cricetomys* giant pouched rat species proposed by Olayemi et al. Tree was constructed on the basis of 2 independent runs, 5 million generations each, based on a 409-bp fragment of the *cytochrome B* gene. Bayesian posterior probabilities for each node are shown. Scale bar indicates nucleotide substitutions per site.

Five of the 9 *Cricetomys* rat sequences obtained in this study were identical (ROC37, ROC38, ROC50, ROC79, and ROC103); we submitted the 5 unique sequences to GenBank (accession nos. MH365330–4). The analyses identified all *Cricetomys* rat specimens as *Cricetomys* Sp3, as previously proposed by Olayemi et al. (Figure 3) (*31*). However, this taxonomy is not currently recognized; therefore these specimens are identified as *C. emini* rats (*34*).

## Discussion

We describe epidemiologic and ecologic investigations of monkeypox in a rural region of the Republic of the Congo. Confirmed and probable monkeypox cases were found in the districts of Enyelle, Impfondo, and Dongou, and possible cases were identified in Betou. There were no epidemiologic links between cases from different districts. All hypothesized human-to-human transmission events appeared to have been contained within the individual districts. In Impfondo, transmission was contained to 1 family with a putative 3 generations of interhuman virus transmission. No evidence of virus introduction from neighboring countries was found.

Although Dongou case-patients 2d and 5d were hospitalized in Impfondo, it appears that case-patient 2d contracted the disease from another person in Manfouété and subsequently transmitted the virus to her mother (case-patient 5d). Although we were unable to link case-patient 5d directly to the other Manfouété cases, Dongou case-patients 6d–14d appear to be part of a family cluster and reported exposure to the same infected individual in Manfouété. Unfortunately, it was not possible to create a transmission chain because the date of symptom onset was not reported for any of these suspected cases. 

MPXV is thought to have limited capacity to spread in human populations, as described by stochastic models (*35*,*36*). We documented 3 suspected serial transmission events; the largest number of previously recorded events is 6 (*21*). Our investigation revealed additional family clusters, suggesting that human-to-human transmission does play a major role in disease transmission.

Most of the suspected cases that could not be further classified were found in Betou, where our investigation did not find any confirmed cases. Additional information would be needed to definitively determine whether monkeypox is actually circulating in this district.

It does appear that independent zoonotic events likely occurred in Enyelle, Impfondo, and Dongou, which are connected by the same forest. These separate events appear to have led to different outbreaks in various parts of Likouala. Although it was previously suspected, our results provide substantial evidence that monkeypox is endemic in this region. Indeed, monkeypox cases have previously been documented in the area, and a 2007 serosurvey found an orthopoxvirus reactive antibody seroprevalence of 56.9% in Likouala Department (*23*).

Although past outbreaks and cases have been observed, the reported disease incidence in Republic of Congo is markedly lower in comparison to the neighboring DRC. Given Impfondo’s close proximity to the Equateur Province in DRC (directly across the Ubangi River), where monkeypox cases are more frequently reported, it is possible that monkeypox was imported from DRC into Republic of Congo, either through human movements or cross-border transportation of bushmeat (*20*). Moreover, these 2 countries have notable biogeographic, ecologic, and cultural similarities, suggesting that observed differences in disease incidence are not necessarily the result of underlying ecology but are perhaps attributable to differences in investment in surveillance. Nevertheless, a deeper exploration of cultural differences, including bushmeat preferences, may be warranted.

This outbreak is indicative of endemic transmission of MPXV, which requires the circulation and maintenance of virus in local mammal populations (*18*,*21*). Although the ecologic study did not result in isolation of live virus in any of the captured animals, 2 (1.9%) of 105 blood/serum specimens were positive for orthopoxvirus IgG. We did not detect viable virus in *Cricetomys* rats, but no data exist regarding how long after infection that virus or viral DNA is detectable in tissue samples. These serologic data support previous published reports (*15*,*37*) that found giant pouched rats could be involved in the circulation and maintenance of MPXV in nature (*31*). Moreover, the taxonomy of the genus *Cricetomys* is currently being debated and possibly revised, which highlights the importance of the correct identification of species to make accurate inferences regarding potential MPXV hosts.

Several limitations were associated with this investigation. Lesion specimens were not collected from most suspected cases; as a result, we are confident only that the 7 confirmed cases are monkeypox. Laboratory confirmation by PCR was not possible for many of the suspect cases because patients were interviewed after the rash had resolved. All interviewed patients did provide blood, but serologic results are insufficient for monkeypox confirmation given the cross-reactivity observed among orthopoxviruses. Furthermore, it is difficult to interpret serologic results and differentiate current infection from past exposure, particularly in the absence of detailed clinical and epidemiologic data. Regardless, the IgM data give us confidence that infection was from recent exposure (within the previous 2 months). However, the likelihood of other rash illness outbreaks, such as measles and chickenpox (caused by varicella zoster virus), during the same period make it difficult to determine the true extent of the outbreak. In addition, missing case report forms and data on available forms limited our ability to classify several cases as probable or possible and to identify chains of transmission across contacts.

In general, monkeypox surveillance in the region needs to be strengthened. The challenges associated with this remote region, such as limited health and transportation infrastructure and the absence of specimen collection supplies and a well-functioning cold chain (system of specimen storage and transport at the recommended cold temperatures), have resulted in inconsistent and incomplete reporting. Therefore, is it difficult to determine the true extent of the outbreak, particularly during a period when other rash illnesses were circulating. We attempted to improve diagnostic capabilities by training healthcare workers to use specimen investigation kits designed to collect direct lesion material rather than blood. In addition, during our training, we found that most healthcare workers who attended had little prior knowledge of monkeypox clinical symptoms, monkeypox case management, and infection control practices. Consistent refresher trainings and additional guidance for monkeypox surveillance will be worthwhile to determine the true burden of monkeypox in this region.
